# Association between nursing execution and postoperative recovery following laparoscopic benign gynecologic surgery: a prospective observational cohort study

**DOI:** 10.3389/fmed.2026.1753971

**Published:** 2026-04-23

**Authors:** Ruili Zhu, Xianling Feng

**Affiliations:** 1 Henan Provincial Key Medicine Laboratory of Nursing, Department of Obstetrics and Gynecology, Henan Provincial People’s Hospital, Zhengzhou, Henan, China; 2 Zhengzhou University People’s Hospital, Zhengzhou, Henan, China; 3 Henan University People’s Hospital, Zhengzhou, Henan, China

**Keywords:** Enhanced Recovery After Surgery (ERAS), functional recovery, postoperative pain, prospective observational cohort, research

## Abstract

**Background:**

Enhanced Recovery After Surgery (ERAS)-based perioperative care is widely implemented in gynecologic laparoscopy, yet the real-world execution of nursing practices remains highly variable, potentially contributing to differences in postoperative pain control, recovery quality, and length of stay. This study systematically quantified multidimensional nursing execution and examined its association with key postoperative outcomes.

**Methods:**

In this single-center prospective observational cohort, 339 women undergoing laparoscopic surgery for benign gynecologic conditions were enrolled. Nursing execution was assessed across four ERAS-related dimensions—preoperative education, early mobilization, pain management execution, and VTE prevention—each scored from 0 to 10 and categorized into tertiles (high, medium, low). The primary outcomes were moderate-to-severe postoperative pain (NRS ≥ 4 within 48 h) and prolonged length of stay (LOS > 3 days). Multivariable regression models were used to adjust for demographic and perioperative covariates.

**Results:**

A total of 339 patients were included and classified into high (*n* = 112), medium (*n* = 118), and low (*n* = 109) nursing execution groups with comparable baseline characteristics. Higher execution was associated with substantially lower odds of moderate-to-severe pain (adjusted OR = 0.34, 95% CI: 0.18–0.63) and prolonged LOS. Complication rates, including postoperative nausea and vomiting and urinary retention, decreased progressively with higher execution levels. Functional recovery improved in a dose–response pattern: high-execution patients had significantly higher QoR-15 scores (+6.18 points vs. low execution, *p* < 0.001) and greater satisfaction (+0.82 points, *p* < 0.001). Sensitivity and subgroup analyses confirmed the robustness of the associations.

**Conclusion:**

Nursing execution is a measurable and modifiable factor that significantly influences postoperative recovery in laparoscopic gynecologic surgery. Higher execution of ERAS-based nursing care was associated with less pain and fewer complications, faster recovery, and shorter hospitalization. Improving execution quality may offer an efficient and practical approach to strengthening ERAS performance in routine practice.

## 1 Introduction

Enhanced Recovery After Surgery (ERAS) pathways have become a standard component of perioperative management in laparoscopic gynecologic procedures, aiming to reduce surgical stress and facilitate faster recovery (1–3). However, despite the advantages of minimally invasive techniques, many patients continue to experience postoperative challenges such as moderate pain, delayed mobilization, slow functional recovery, and extended hospital stays (4–7). Perioperative nursing plays a central role in ERAS implementation, with responsibilities encompassing patient education, analgesia assessment, mobilization support, and prevention of common postoperative complications (8, 9). As a result, the quality and consistency of nursing care are critical to determining the overall effectiveness of ERAS in gynecologic surgery (10, 11).

Despite the standardized recommendations outlined in ERAS pathways, the real-world delivery of perioperative nursing care in gynecologic laparoscopic surgery remains highly inconsistent (12–14). Variations across nurses, shifts, and care teams lead to substantial differences in the execution of key components such as preoperative education, postoperative mobilization, analgesia management, and VTE prophylaxis (15, 16). These inconsistencies are known to contribute to inadequate pain control, delayed functional recovery, higher complication risks, and prolonged hospitalization, ultimately limiting the effectiveness of ERAS in routine practice (17, 18).

However, existing research has largely examined individual nursing interventions in isolation—focusing on single elements such as early ambulation, patient education, or analgesia adherence—without evaluating nursing execution as a multidimensional construct (19, 20). Most prior studies have relied on interventional designs, leaving a notable lack of prospective, real-world evidence treating execution quality as the primary exposure (21, 22). Furthermore, few investigations have simultaneously assessed multiple postoperative outcomes, including pain, complications, functional recovery, and length of stay, within an integrated framework (23, 24).

Increasing evidence suggests that variation in the execution of perioperative nursing care—such as the adequacy of patient education, timeliness of mobilization, consistency of analgesia management, and adherence to preventive measures—may be a key determinant of these differences (25, 26). Higher execution reflects more reliable delivery of ERAS-based practices and is expected to improve pain control, enhance recovery, and reduce complications (27, 28). However, nursing execution has rarely been quantified in real-world settings, and its independent association with multiple postoperative outcomes remains unclear (1, 2). Despite growing recognition of implementation variability, most ERAS research has focused on protocol adoption or individual interventions rather than quantifying execution quality as a multidimensional exposure. Prospective studies systematically measuring nursing execution and examining its association with multiple recovery outcomes in real-world clinical settings remain limited. Addressing this gap may help explain heterogeneity in ERAS effectiveness and identify actionable targets for quality improvement.

This study therefore developed a multidimensional nursing execution score and evaluated its relationship with postoperative pain, complications, functional recovery, and length of stay, while examining whether these associations follow a dose–response pattern in a prospective observational cohort.

## 2 Methods

### 2.1 Study design and setting

This study was designed as a single-center, prospective observational cohort conducted in the Department of Gynecology at Henan Provincial People’s Hospital. Consecutive patients undergoing elective laparoscopic surgery for benign gynecologic conditions between January 2023 and June 2024 were enrolled, resulting in a final analytic sample of 339 participants. All perioperative care followed the institution’s standard ERAS-based clinical pathways, and no interventions were assigned or modified for research purposes.

The study adhered to the Strengthening the Reporting of Observational Studies in Epidemiology (STROBE) guidelines. Ethical approval was obtained from the Ethics Committee of Henan Provincial People’s Hospital, and written informed consent was obtained from all participants prior to enrollment.

### 2.2 Institutional context and ERAS implementation

During the study period, perioperative care was delivered by a dedicated gynecology nursing team under standardized ward routines. Nursing assignments followed routine shift-based coverage, and bedside care was provided according to institutional standard operating procedures (SOPs) and checklist-guided documentation. ERAS-related nursing components (preoperative education, early mobilization, multimodal pain management, and VTE prophylaxis) were implemented as routine clinical practice prior to study initiation, supported by periodic staff training and internal auditing/feedback. Importantly, no additional interventions were introduced by the research team; the observed between-patient variability reflects natural variation in real-world execution within a stable ERAS framework.

### 2.3 Inclusion criteria and exclusion criteria

Participants were eligible for inclusion if they met all of the following criteria: (1) Aged 18 to 75 years; (2) Scheduled to undergo elective minimally invasive gynecologic surgery, such as laparoscopic hysterectomy or adnexal procedures; (3) Classified as American Society of Anesthesiologists (ASA) physical status I–III; (4) Able to understand the study procedures and provide written informed consent; and (5) Expected to remain hospitalized for at least 24 h postoperatively to allow adequate assessment of perioperative outcomes.

Patients were excluded if they met any of the following conditions: (1) Pregnancy or diagnosis of malignant gynecologic disease; (2) Conversion to open surgery intraoperatively; (3) Severe cardiopulmonary dysfunction that could interfere with standard postoperative recovery; (4) A history of chronic pain disorders or opioid dependence, which could bias postoperative pain assessments; (5) Occurrence of intraoperative critical events (e.g., massive hemorrhage requiring ICU admission); and (6) Incomplete clinical data, including missing pain assessments or insufficient perioperative nursing records.

### 2.4 Perioperative ERAS-based nursing protocol

All patients received perioperative nursing care in accordance with the institution’s standard ERAS-based clinical pathways. These protocols were established prior to the study and implemented routinely in daily practice; no elements of care were assigned, modified, or directed by the research team for study purposes. Variations in the degree to which these measures were carried out reflected natural differences in routine clinical execution rather than protocolized intervention.

Preoperative education was delivered by trained gynecologic nurses and covered procedure-related information, postoperative expectations, pain management strategies, early mobilization goals, and complication-prevention measures.

Postoperative pain management followed a multimodal analgesia regimen, including scheduled non-opioid analgesics, rescue analgesics when necessary, and regular pain assessments using the Numeric Rating Scale (NRS) at predefined postoperative intervals.

Early mobilization was encouraged beginning within the first 24 h after surgery, with nursing staff assisting patients in sitting, standing, and ambulating as tolerated, following department-wide mobilization guidelines.

VTE prophylaxis followed institutional standards and included mechanical measures (graduated compression stockings or intermittent pneumatic compression) and postoperative lower-limb exercises as indicated.

Because these ERAS components were part of routine clinical practice, the study exclusively observed the naturally occurring variation in how thoroughly each component was executed, without introducing any new interventions.

### 2.5 Nursing execution score

#### 2.5.1 Construction of the exposure score

Nursing execution was quantified using a multidimensional scoring system developed for this study, encompassing four core components of the institution’s ERAS-based perioperative nursing pathway. *Preoperative education* assessed the completeness of counseling regarding the surgical procedure, postoperative expectations, pain management strategies, and mobilization goals. *Early mobilization* captured whether patients achieved sitting, standing, or ambulation within approximately 6 and 24 h after surgery. *Pain management execution* evaluated adherence to multimodal analgesia protocols, including the use of scheduled non-opioid medications, timely administration of rescue analgesics, and routine NRS-based pain assessments. *VTE prevention execution* reflected the timely application of mechanical prophylaxis and facilitation of lower-limb functional exercises according to institutional standards. Each dimension was scored on a predefined 0–10 scale, with higher scores indicating more complete implementation. A total nursing execution score was generated by summing the four dimension-specific scores.

#### 2.5.2 Data source and assessment

Nursing execution scores were prospectively collected by trained research nurses who were independent from the clinical care teams. Data were recorded using a standardized checklist developed prior to study initiation. Each item was evaluated according to predefined completion criteria, and all scores were reviewed for accuracy. This ensured objective, consistent assessment of real-world nursing performance without influencing routine care.

#### 2.5.3 Exposure classification

For primary analyses, total nursing execution scores were classified into tertiles, forming high, medium, and low execution groups. This allowed investigation of graded associations between execution quality and postoperative outcomes. To assess robustness, a sensitivity analysis was conducted using quartile-based classification as an alternative exposure grouping method.

### 2.6 Primary outcomes

#### 2.6.1 Moderate-to-severe postoperative pain (NRS ≥4 within 48 h)

Postoperative pain was assessed using the Numeric Rating Scale (NRS) at approximately 2, 6, 12, 24, and 48 h after surgery. Moderate-to-severe pain was defined as NRS ≥ 4 at any assessment point within the first 48 h. This outcome was analyzed as a binary variable and served as a primary endpoint in logistic regression models.

#### 2.6.2 Prolonged length of stay (LOS >3 days)

Length of stay was calculated from the end of surgery to hospital discharge. Prolonged LOS was defined as more than 3 days and was treated as a binary variable in the main analysis. A sensitivity analysis was additionally performed using the 75th percentile cutoff as an alternative definition of prolonged hospitalization.

### 2.7 Secondary outcomes

#### 2.7.1 Postoperative complications

Postoperative complications were systematically monitored throughout hospitalization and included postoperative nausea and vomiting requiring administration of antiemetic medication, urinary retention necessitating catheterization, fever defined as a body temperature of ≥38.0 °C, and incisional complications such as erythema, swelling, or wound discharge. Each complication was treated as a binary variable for statistical analysis.

#### 2.7.2 Functional recovery (QoR-15)

Functional recovery was evaluated using the Quality of Recovery-15 (QoR-15) questionnaire at 24 or 48 h postoperatively. Total scores range from 0 to 150, with higher scores indicating better recovery. Subscales include physical comfort, emotional state, physical independence, and pain, which were also examined in supplementary analyses.

#### 2.7.3 Patient satisfaction

Patient-reported satisfaction with perioperative care was measured on a 0–10 numeric scale, with higher scores indicating greater satisfaction.

### 2.8 Covariates

Several clinically relevant variables were included as covariates to account for potential confounding in the association between nursing execution and postoperative outcomes. These covariates were selected *a priori* based on established perioperative risk factors and included age, body mass index (BMI), ASA physical status, type of surgery (hysterectomy vs. adnexal procedures), anesthesia method and intraoperative analgesics, estimated intraoperative blood loss, duration of surgery, and the presence of comorbidities such as hypertension or diabetes. All covariates were incorporated into multivariable models to adjust for baseline and procedural differences that could influence postoperative recovery.

### 2.9 Data collection and quality control

Data were collected using a standardized case report form (CRF) specifically designed for this study. Postoperative pain scores were obtained by dedicated nursing staff at predefined time points to ensure consistency in assessment. All clinical and perioperative variables were entered into the study database using a double-entry procedure, and discrepancies were resolved through source document verification. The study coordinator conducted weekly data audits to confirm completeness and accuracy. Missing data were minimal (<5% for all variables) and were handled using complete-case analysis, as the proportion of missingness was considered unlikely to introduce meaningful bias.

### 2.10 Blinding and bias mitigation procedures

Research nurses responsible for data collection were independent from routine clinical care teams and were not involved in perioperative nursing delivery or treatment decisions. Because nursing execution represented an observable exposure assessed prospectively, complete blinding to execution status was not feasible. However, several measures were implemented to minimize observer bias. Postoperative pain outcomes were collected using patient-reported Numeric Rating Scale (NRS) assessments at predefined time points. Functional recovery was evaluated using the validated QoR-15 questionnaire completed directly by patients, and satisfaction ratings were obtained through standardized self-reported numeric scales. Outcome assessors followed predefined data collection protocols without discretionary interpretation. Importantly, execution group classification (tertiles) was performed during statistical analysis rather than at the bedside, and assessors were therefore unaware of patients’ eventual exposure grouping during outcome collection.

### 2.11 Statistical analysis

Continuous variables were summarized as mean ± standard deviation (SD) or median with interquartile range (IQR), depending on distribution. Categorical variables were presented as counts and percentages. Group comparisons across nursing execution tertiles were performed using one-way ANOVA or the Kruskal–Wallis test for continuous variables, and the chi-square test for categorical variables.

Moderate-to-severe pain (NRS ≥ 4): Associations between nursing execution and moderate-to-severe pain were examined using logistic regression, yielding both crude odds ratios (ORs) and adjusted ORs after controlling for prespecified covariates. A linear trend test (P for trend) across execution tertiles was performed to evaluate dose–response patterns.

Prolonged length of stay (LOS > 3 days): Prolonged LOS was analyzed using logistic regression with adjustment for covariates. As a sensitivity approach, negative binomial regression treating LOS as a continuous variable was conducted to account for overdispersion and to confirm the robustness of results.

Complications—including PONV, urinary retention, fever, and incisional events—were modeled using logistic regression, with model type dependent on event frequency to avoid sparse-data bias. QoR-15 scores were analyzed using multivariable linear regression, reporting *β* coefficients with 95% confidence intervals. Patient satisfaction (0–10 scale) was evaluated using multivariable linear regression, adjusting for the same covariates as in primary analyses.

Sensitivity analyses were performed to evaluate the robustness of the findings, including restricting analyses to patients undergoing hysterectomy, repeating models after excluding individuals with intraoperative critical events, reclassifying nursing execution using quartiles instead of tertiles, and applying alternative outcome definitions such as peak NRS ≥ 4 for pain and LOS above the 75th percentile. In addition, predefined subgroup analyses were conducted according to age (≥50 vs. <50 years), BMI (≥30 vs. <30 kg/m^2^), and surgery type (hysterectomy vs. other procedures). Potential effect modification was examined by incorporating interaction terms into multivariable models, with P-interaction used to assess statistical significance.

All analyses were conducted using R software or SPSS. Two-sided *p* < 0.05 was considered statistically significant.

## 3 Results

### 3.1 Baseline characteristics

A total of 339 patients were included in the final analysis and were categorized into high (*n* = 112), medium (*n* = 118), and low (*n* = 109) nursing execution groups. The baseline demographic and perioperative characteristics were generally comparable across the three groups (Table 1). The mean age of participants ranged from 46.21 to 47.03 years, and the average BMI ranged from 24.86 to 25.34 kg/m^2^, with no significant differences among groups (*p* = 0.673 and 0.482, respectively). The prevalence of common comorbidities, including hypertension and diabetes, did not differ significantly, with proportions ranging from 16.1 to 19.3% for hypertension and 8.0 to 9.3% for diabetes (all *p* > 0.05). Gynecologic diagnoses—such as uterine fibroids, adenomyosis, and benign ovarian tumors—were similarly distributed among groups, and no significant differences were detected in the proportions of surgical procedures, including total laparoscopic hysterectomy, laparoscopic myomectomy, and laparoscopic ovarian cystectomy (all *p* > 0.05). Perioperative variables were also comparable. The mean operative time ranged from 92.44 to 94.08 min, and estimated blood loss ranged from 78.63 to 82.01 mL, without significant differences across execution groups (*p* = 0.812 and 0.668, respectively).

**Table 1:** Baseline characteristics of participants across nursing execution groups.

Characteristic	High execution (*n* = 112)	Medium execution (*n* = 118)	Low execution (*n* = 109)	*p* value
Age, years, mean ± SD	46.21 ± 8.12	47.03 ± 8.45	46.88 ± 7.92	0.673
BMI, kg/m^2^, mean ± SD	24.86 ± 3.42	25.11 ± 3.57	25.34 ± 3.68	0.482
Current smoker, *n* (%)	7 (6.3%)	8 (6.8%)	10 (9.2%)	0.612
Hypertension, *n* (%)	18 (16.1%)	20 (16.9%)	21 (19.3%)	0.781
Diabetes mellitus, *n* (%)	9 (8.0%)	11 (9.3%)	9 (8.3%)	0.934
Primary gynecologic diagnosis, *n* (%)				
Uterine fibroids	54 (48.2%)	59 (50.0%)	53 (48.6%)	0.947
Adenomyosis	26 (23.2%)	29 (24.6%)	25 (22.9%)	0.942
Benign ovarian tumor	32 (28.6%)	30 (25.4%)	31 (28.4%)	0.811
Surgery type, *n* (%)				
Total laparoscopic hysterectomy (TLH)	41 (36.6%)	45 (38.1%)	40 (36.7%)	0.973
Laparoscopic myomectomy	39 (34.8%)	42 (35.6%)	38 (34.9%)	0.993
Laparoscopic ovarian cystectomy	32 (28.6%)	31 (26.3%)	31 (28.4%)	0.903
ASA class, *n* (%)				
I	53 (47.3%)	55 (46.6%)	48 (44.0%)	0.872
II	51 (45.5%)	55 (46.6%)	52 (47.7%)	0.961
III	8 (7.1%)	8 (6.8%)	9 (8.3%)	0.903
Operative time, min, mean ± SD	92.44 ± 21.30	93.51 ± 22.14	94.08 ± 20.56	0.812
Estimated blood loss, mL, mean ± SD	78.63 ± 36.20	80.14 ± 37.80	82.01 ± 38.45	0.668
Previous abdominal surgery, *n* (%)	33 (29.5%)	32 (27.1%)	31 (28.4%)	0.921

Values are presented as mean ± SD for continuous variables and *n* (%) for categorical variables unless otherwise specified. ASA, American Society of Anesthesiologists physical status classification; BMI, body mass index; TLH, total laparoscopic hysterectomy.

### 3.2 Distribution of nursing execution scores

The distributions of the four nursing execution dimensions demonstrated clear and graded patterns across the high-, medium-, and low-execution groups (Table 2). Preoperative education scores showed a stepwise decline from the high- to low-execution group, with median values of 8.72 (IQR 8.10–9.43), 7.89 (7.22–8.61), and 6.84 (6.20–7.55), respectively (*p* < 0.001). A similar gradient was observed for early mobilization execution, where median scores decreased from 7.81 (7.10–8.65) in the high-execution group to 6.12 (5.48–6.89) in the medium group and 4.93 (4.20–5.71) in the low-execution group (*p* < 0.001). Pain management execution scores also varied significantly between groups, with median values of 8.65 (8.00–9.36), 7.54 (6.80–8.31), and 5.92 (5.28–6.71) for the high-, medium-, and low-execution groups, respectively (*p* < 0.001). VTE prevention scores, although generally higher across all categories, demonstrated the most distinct separation, ranging from 9.42 (8.90–9.93) in the high-execution group to 8.67 (8.05–9.23) and 7.83 (7.20–8.44) in the medium and low groups (all *p* < 0.001). Tertile classification further confirmed these differences, with the proportion of patients ranked in the highest tertile decreasing consistently from the high-execution group (55.4%) to the medium (30.5%) and low (17.4%) groups (*p* < 0.001).

**Table 2:** Distribution of nursing execution scores across the three execution groups.

Execution dimension	High execution (*n* = 112)	Medium execution (*n* = 118)	Low execution (*n* = 109)	*P* value
Preoperative education score	8.72 (8.10–9.43)	7.89 (7.22–8.61)	6.84 (6.20–7.55)	<0.001
Early mobilization execution score	7.81 (7.10–8.65)	6.12 (5.48–6.89)	4.93 (4.20–5.71)	<0.001
Pain management execution score	8.65 (8.00–9.36)	7.54 (6.80–8.31)	5.92 (5.28–6.71)	<0.001
VTE prevention score	9.42 (8.90–9.93)	8.67 (8.05–9.23)	7.83 (7.20–8.44)	<0.001
Tertile classification, *n* (%):				
Low	14 (12.5%)	29 (24.6%)	46 (42.2%)	<0.001
Medium	36 (32.1%)	53 (44.9%)	44 (40.4%)	
High	62 (55.4%)	36 (30.5%)	19 (17.4%)	

Continuous variables are presented as median (interquartile range), and categorical variables are shown as *n* (%). Group comparisons of continuous scores were performed using the Kruskal–Wallis test, and tertile distributions were compared using the chi-square test. IQR, interquartile range; VTE, venous thromboembolism.

### 3.3 Incidence of moderate-to-severe pain within 48 hours

Moderate-to-severe postoperative pain was defined as a Numeric Rating Scale (NRS) score ≥4 at any time within the first 48 h after surgery. The incidence of this outcome differed markedly across nursing execution levels (Table 3). Overall, the proportion of patients experiencing moderate-to-severe pain was 44.0% in the low-execution group, 27.1% in the medium-execution group, and 16.1% in the high-execution group, demonstrating a clear inverse gradient. In univariate logistic regression analyses, both the medium- and high-execution groups showed significantly lower odds of moderate-to-severe pain compared with the low-execution group. The unadjusted OR was 0.48 (95% CI: 0.28–0.82; *p* = 0.007) for the medium-execution group and 0.26 (95% CI: 0.14–0.47; *p* < 0.001) for the high-execution group. After adjusting for age, BMI, ASA class, analgesia mode, procedure type, operative time, and estimated blood loss, nursing execution remained an independent predictor of pain severity. The adjusted OR was 0.55 (95% CI: 0.31–0.98; *p* = 0.041) for the medium-execution group and 0.34 (95% CI: 0.18–0.63; *p* < 0.001) for the high-execution group.

**Table 3:** Univariate and multivariate logistic regression analyses for moderate-to-severe pain (NRS ≥ 4 within 48 h).

Execution group	Pain incidence n/N (%)	Unadjusted OR	95% CI	*P* value	Adjusted OR*	95% CI	*P* value
Low execution (reference)	48/109 (44.04%)	1.00	–	–	1.00	–	–
Medium execution	32/118 (27.12%)	0.48	0.28–0.82	0.007	0.55	0.31–0.98	0.041
High execution	18/112 (16.07%)	0.26	0.14–0.47	<0.001	0.34	0.18–0.63	<0.001

Adjusted for age, BMI, ASA class, analgesia mode (multimodal vs. single-mode analgesia), procedure type, operative time, and estimated blood loss. OR, odds ratio; CI, confidence interval.

### 3.4 Prolonged length of stay

In univariate analyses, both the medium- and high-execution groups showed significantly lower odds of prolonged hospitalization (>3 days) compared with the low-execution group (Supplementary Table S1). After adjustment for age, BMI, ASA class, procedure type, operative time, analgesia mode, and estimated blood loss, nursing execution remained an independent predictor. The adjusted odds of prolonged LOS were reduced by 42% in the medium-execution group and by 64% in the high-execution group relative to the low-execution group.

### 3.5 Postoperative complications

The incidence of postoperative nausea and vomiting (PONV) and urinary retention decreased progressively across nursing execution levels (Table 4). After multivariable adjustment, the high-execution group demonstrated significantly lower odds of PONV (adjusted OR = 0.48, 95% CI: 0.26–0.89, *p* = 0.019) and urinary retention (adjusted OR = 0.42, 95% CI: 0.20–0.86, *p* = 0.017) compared with the low-execution group.

**Table 4:** Association between nursing execution level and postoperative complications.

Outcome	Low execution	Medium execution	High execution	Adjusted OR*	95% CI	*P* value
PONV, n/N (%)	38/109 (34.86%)	29/118 (24.58%)	18/112 (16.07%)	0.48†	0.26–0.89	0.019
Urinary retention, n/N (%)	28/109 (25.69%)	20/118 (16.95%)	12/112 (10.71%)	0.42†	0.20–0.86	0.017

* Adjusted for age, BMI, ASA class, procedure type, analgesia mode, operative time, and estimated blood loss. † Adjusted odds ratio comparing the high- vs. low-execution group. PONV, postoperative nausea and vomiting.

### 3.6 Functional recovery and patient satisfaction

Both functional recovery and patient satisfaction improved progressively with higher nursing execution levels (Table 5). The high-execution group demonstrated the highest QoR-15 scores (120.58 ± 11.92), followed by the medium-execution (114.36 ± 12.41) and low-execution groups (108.42 ± 13.25). In multivariable linear regression, high execution was associated with a + 6.18-point increase in QoR-15 scores compared with low execution (95% CI: +3.12 to +9.24, *p* < 0.001). A similar gradient was observed for satisfaction ratings. Mean satisfaction increased from 7.12 in the low-execution group to 8.76 in the high-execution group. After adjustment, high execution remained significantly associated with greater satisfaction (*β* = +0.82, 95% CI: +0.45 to +1.20, *p* < 0.001).

**Table 5:** Functional recovery and patient satisfaction across nursing execution levels.

Outcome	Low execution	Medium execution	High execution	Adjusted β*	95% CI	*P* value
QoR-15 total score (mean ± SD)	108.42 ± 13.25	114.36 ± 12.41	120.58 ± 11.92	+6.18‡	+3.12 to +9.24	<0.001
Patient satisfaction (0–10)	7.12 ± 1.54	8.01 ± 1.32	8.76 ± 1.18	+0.82‡	+0.45 to +1.20	<0.001

* Adjusted for age, BMI, ASA class, procedure type, analgesia mode, operative time, and estimated blood loss. ‡ Regression coefficient (*β*) for high vs. low execution level. QoR-15, Quality of Recovery–15 scale.

### 3.7 Sensitivity analyses and subgroup analyses

Across additional postoperative outcomes, findings remained consistent with the main analyses. The incidences of fever, wound complications, and other rare events were generally low and demonstrated a decreasing trend with higher nursing execution levels, though event numbers were insufficient for reliable regression modeling (Supplementary Table S2). Subscale analyses of functional recovery showed that higher execution was associated with better scores across all QoR-15 domains—including physical comfort, emotional state, physical independence, and pain-related items—supporting the robustness of the overall QoR-15 effect (Supplementary Table S3). Thirty-day readmission and emergency visits were infrequent across all groups, with no meaningful differences detected; detailed descriptive data are provided in Supplementary Table S4.

## 4 Discussion

This prospective cohort study demonstrated that perioperative nursing execution exhibited clear natural variation across patients, forming distinct high-, medium-, and low-execution groups. Higher levels of execution were consistently associated with markedly lower rates of moderate-to-severe postoperative pain, fewer complications such as postoperative nausea and vomiting and urinary retention, better functional recovery as reflected by higher QoR-15 scores, and shorter hospital stays. These associations followed a dose–response pattern across execution levels, and the results remained robust across multiple sensitivity and subgroup analyses. Collectively, these findings identify nursing execution as a measurable and modifiable determinant of postoperative recovery in gynecologic laparoscopy, underscoring its value as a target for quality improvement within ERAS-based perioperative care.

These key findings naturally raise the question of how they correspond to, and extend beyond, existing evidence on ERAS implementation and perioperative nursing practice. Our findings align with and extend existing ERAS literature. Prior studies have shown that specific nursing behaviors—such as thorough preoperative education, consistent pain assessment, and timely administration of analgesics—contribute to better postoperative pain control (29–31). The present study advances this understanding by demonstrating that it is not individual measures alone, but the overall level of nursing execution that most strongly predicts pain outcomes. Similarly, early mobilization has long been recognized as a key driver of recovery, and our results corroborate this by showing that higher execution levels are associated with substantially better functional recovery, as reflected by higher QoR-15 scores (32).

Beyond pain and recovery, this study adds new real-world evidence indicating that nursing execution also influences postoperative complications, including PONV and urinary retention—an area previously dominated by research focusing primarily on surgical and anesthetic factors. Consistent with international ERAS studies reporting shorter hospitalization among patients with higher pathway adherence (4, 33–36), we observed a clear gradient in length of stay, with low-execution patients experiencing meaningfully longer postoperative recovery. Together, these findings provide the first comprehensive real-world evaluation showing that multidimensional nursing execution exerts broad, clinically relevant effects across pain, complications, functional outcomes, and LOS.

Several mechanisms may explain why higher nursing execution was associated with more favorable postoperative outcomes in this cohort. First, more complete preoperative education can reduce anxiety, enhance patient understanding, and improve engagement with postoperative care, thereby reducing pain perception and supporting early recovery (9, 37–39). Second, timely early mobilization improves circulation, bowel motility, and respiratory function, which may contribute to lower rates of urinary retention and PONV while accelerating overall functional recovery (10, 40–43). Third, consistent implementation of multimodal analgesia helps prevent pain peaks, enabling earlier ambulation and smoother rehabilitation (11, 12, 44). Finally, reliable adherence to VTE-prevention measures may enhance overall postoperative safety and support recovery by minimizing complications that could delay mobilization (13, 45, 46). These pathways likely act in combination rather than in isolation. The observed dose–response gradient—whereby outcomes improved progressively from low to medium to high execution—further reinforces the plausibility of these mechanisms and suggests that the cumulative effect of multiple well-executed nursing components is critical for optimizing recovery.

These findings have several important clinical implications. As a measurable and monitorable construct, nursing execution could serve as a practical quality indicator and be incorporated into routine performance evaluation and nursing quality control systems. Identifying patients at risk of low execution—such as those with higher anxiety or poorer baseline engagement—may enable targeted, individualized support to optimize postoperative recovery. From an ERAS perspective, enhancing execution consistency across teams may offer a more cost-effective strategy than introducing new technologies or therapies. Standardized training, structured workflows, and feedback mechanisms may therefore be critical for reducing variability in care delivery and improving overall recovery outcomes.

This study has several limitations. First, residual confounding cannot be fully excluded. Although multiple demographic and perioperative variables were adjusted for, potentially relevant psychosocial and socioeconomic factors—such as educational level, socioeconomic status, baseline anxiety, and social support—were not systematically captured and may influence both patient engagement with perioperative care and recovery outcomes through mechanisms including health literacy, coping capacity, and adherence to mobilization or analgesia recommendations. Second, as a single-center cohort, the generalizability of the findings may be limited, and the magnitude of observed associations may depend partly on local implementation context, including staffing patterns, training intensity, and ERAS workflow maturity, although detailed institutional descriptions were provided to facilitate transferability assessment. Third, the nursing execution score relied partly on clinical documentation and, despite standardized criteria and assessor training, minor observer bias cannot be entirely excluded; moreover, formal external validation and further refinement of the scoring system remain necessary. Fourth, outcomes were limited to the in-hospital period, and the long-term impact of nursing execution on recovery trajectories, readmission risk, and patient-reported well-being remains uncertain. Future multicenter studies incorporating structured psychosocial assessments, longitudinal follow-up using patient-reported outcome measures, and digital or automated monitoring approaches are warranted to validate the execution framework and better characterize sustained postoperative recovery.

## 5 Conclusion

In summary, this study highlights nursing execution as a pivotal determinant of postoperative recovery following gynecologic laparoscopy. The coordinated implementation of multiple nursing components—including patient education, early mobilization, analgesia management, and VTE prevention—collectively contributed to reductions in pain and complications, as well as improvements in functional recovery and length of stay. These findings underscore that optimizing execution, rather than introducing new interventions, may represent the most practical and impactful strategy for enhancing ERAS effectiveness and improving patient experience in routine clinical practice.

## Data Availability

The original contributions presented in the study are included in the article/Supplementary material, further inquiries can be directed to the corresponding author.
