# Supplementary material for: Association between nursing execution and postoperative recovery following laparoscopic benign gynecologic surgery: a prospective observational cohort study
Source: Front Med (Lausanne). 2026 Apr 23;13:1753971. doi: 10.3389/fmed.2026.1753971 (PMC13149466; doi:10.3389/fmed.2026.1753971)
Supplement: Supplementary file 1 [file Data_Sheet_1.docx]

**Supplementary Table S1. Univariate and Multivariate Logistic Regression for Prolonged LOS (>3 days)**

| **Execution Level** | **LOS >3d n/N (%)** | **Unadjusted OR** | **95% CI** | **P value** | **Adjusted OR*** | **95% CI** | **P value** |
| --- | --- | --- | --- | --- | --- | --- | --- |
| Low (ref) | 42/109 (38.53%) | 1.00 | — | — | 1.00 | — | — |
| Medium | 26/118 (22.03%) | 0.45 | 0.26–0.78 | 0.004 | 0.58 | 0.34–0.98 | 0.042 |
| High | 14/112 (12.50%) | 0.23 | 0.12–0.43 | <0.001 | 0.36 | 0.20–0.65 | <0.001 |

**Notes:**
*Adjusted for age, BMI, ASA class, procedure type, operative time, analgesia mode, and estimated blood loss.
OR = odds ratio; CI = confidence interval.*

**Supplementary Table S2. Additional Postoperative Complications (Fever, Wound Events, Rare Events)**

| **Complication** | **Low Execution** | **Medium Execution** | **High Execution** | **Adjusted OR*** | **95% CI** | **P value** |
| --- | --- | --- | --- | --- | --- | --- |
| **Fever ≥38°C, n/N (%)** | 14 / 109 (12.84%) | 9 / 118 (7.63%) | 6 / 112 (5.36%) | 0.55 | 0.25–1.20 | 0.134 |
| **Wound complications, n/N (%)** | 6 / 109 (5.50%) | 4 / 118 (3.39%) | 2 / 112 (1.79%) | 0.47 | 0.13–1.48 | 0.182 |
| **Rare events†, n/N (%)** | 3 / 109 (2.75%) | 1 / 118 (0.85%) | 1 / 112 (0.89%) | — | — | — |

**Notes:**
*Adjusted for age, BMI, ASA, analgesia mode, procedure type, operative time, and estimated blood loss.
†Rare events include mild allergic reactions, transient hypoxia episodes, or medication intolerance; event numbers were insufficient for regression modeling.

**Supplementary Table S3. QoR-15 Subscale Scores Across Nursing Execution Levels**

| **QoR-15 Subscale** | **Low Execution (mean ± SD)** | **Medium Execution** | **High Execution** | **Adjusted β*** | **95% CI** | **P value** |
| --- | --- | --- | --- | --- | --- | --- |
| **Physical comfort** | 38.52 ± 5.86 | 41.18 ± 5.42 | 44.09 ± 5.01 | +2.12 | +0.90 to +3.35 | 0.001 |
| **Emotional state** | 27.84 ± 4.96 | 29.63 ± 4.70 | 31.42 ± 4.58 | +1.34 | +0.55 to +2.12 | 0.001 |
| **Physical independence** | 17.36 ± 3.38 | 18.42 ± 3.10 | 19.81 ± 2.97 | +1.06 | +0.32 to +1.80 | 0.006 |
| **Pain-related items** | 24.70 ± 4.28 | 25.98 ± 4.10 | 27.61 ± 3.84 | +1.25 | +0.35 to +2.15 | 0.007 |

**Notes:**
*Adjusted for age, BMI, ASA class, procedure type, analgesia mode, operative time, and estimated blood loss.
Higher scores indicate better recovery.

**Supplementary Table S4. Thirty-Day Readmission and Emergency Visits**

| **Outcome** | **Low Execution** | **Medium Execution** | **High Execution** | **Adjusted OR*** | **95% CI** | **P value** |
| --- | --- | --- | --- | --- | --- | --- |
| **30-day readmission, n/N (%)** | 3 / 109 (2.75%) | 2 / 118 (1.69%) | 1 / 112 (0.89%) | —† | — | — |
| **30-day ED visit, n/N (%)** | 4 / 109 (3.67%) | 3 / 118 (2.54%) | 2 / 112 (1.79%) | —† | — | — |

**Notes:**
*Adjustment not performed due to very low event numbers.
†Regression modeling not feasible; outcomes described descriptively only.
